# Extracting Cross-Sectional Clinical Images Based on Their Principal Axes of Inertia

**DOI:** 10.1155/2017/1468596

**Published:** 2017-12-19

**Authors:** Yuzhou Fan, Liangping Luo, Marija Djuric, Zhiyu Li, Djordje Antonijevic, Petar Milenkovic, Yueyang Sun, Ruining Li, Yifang Fan

**Affiliations:** ^1^School of Physical Education and Sport Science, Fujian Normal University, Fuzhou 350117, China; ^2^Shenzhen Tourism College, Jinan University, Guangzhou 518053, China; ^3^Medical Imaging Center, The First Affiliated Hospital of Jinan University, Guangzhou 510632, China; ^4^Laboratory for Anthropology, Institute of Anatomy, School of Medicine, University of Belgrade, 11000 Belgrade, Serbia; ^5^College of Foreign Studies, Jinan University, Guangzhou 510632, China; ^6^Institute for Oncology and Radiology of Serbia, University of Belgrade, 11000 Belgrade, Serbia

## Abstract

Cross-sectional imaging is considered the gold standard in diagnosing a range of diseases. However, despite its widespread use in clinical practice and research, no widely accepted method is available to reliably match cross-sectional planes in several consecutive scans. This deficiency can impede comparison between cross-sectional images and ultimately lead to misdiagnosis. Here, we propose and demonstrate a method for finding the same imaging plane in images obtained during separate scanning sessions. Our method is based on the reconstruction of a “virtual organ” from which arbitrary cross-sectional images can be extracted, independent of the axis orientation in the original scan or cut; the key is to establish unique body coordinates of the organ from its principal axes of inertia. To verify our method a series of tests were performed, and the same cross-sectional plane was successfully extracted. This new approach offers clinicians access, after just a single scanning session, to the morphology and structure of a lesion through cross-sectional images reconstructed along arbitrary axes. It also aids comparable detection of morphological and structural changes in the same imaging plane from scans of the same patient taken at different times—thus potentially reducing the misdiagnosis rate when cross-sectional images are interpreted.

## 1. Introduction

*In vivo* and* ex vivo *cross section image (CSI) (defined here as encompassing magnetic resonance imaging (MRI), computed tomography (CT), and any images of serial sections of human organ) is taking an increasingly important role in medicine. Clinically, it is considered to be the gold standard for the diagnosis of a wide range of diseases [1–3]. Scientifically, the analysis of CSIs is an essential step in a broad range of studies [4–9]. In particular, many studies [10–14] have shown that the analysis of CSIs is of key importance when describing and predicting morphological changes in organs.

In order to use CSIs for medical examination, reliable methods for determining the anatomical coordinate system, the anatomical landmarks and their relationship are required. Since 1750 when Euler first discovered and defined principal axes of inertia (PAI), it has been used to establish a body coordinate system for different methods [15–21]. Recently, PAI has been used to define an automated patient-specific anatomical coordinate system for the distal femur and proximal tibia [22], to characterize the geometry of the carpal bones [23], to investigate the rotational variations of temporomandibular joint trajectories [24], to measure the anisotropy of mandible's trabecular bone [25], and to calculate the bone stresses adjacent to dental implants [26].

Despite such a widespread use of CSI, the approach, as practiced today, has critical limitations. For example, a recent analysis of 117,348 imaging orders from 3340 clinicians showed that computerized decision-support systems failed in two out of three cases to match selected MRI, CT, and nuclear-medicine based imaging procedures with the appropriate criteria [27]. If we directly use the CSIs obtained in different scanning session, the resulting diagnosis may not be reliable because it is very difficult for patients to maintain the same position, even with the help of the stereotactic frames. Also, scans are often taken with the participants positioned in the standard anatomical posture, but when the interval between two scans is long, reproducing the patient's previous posture is near-impossible. Moreover, other factors such as different radiographers, different CT scanner systems, physical changes of the participant, and even breathing can all make it difficult to resume the identical posture [28, 29].

Although the analysis of CSIs using anatomical coordinate system based on PAI and center of mass (COM) determination has been remarkably employed, a solution to the problem of matching cross-sectional planes extracted from “virtual organs” reconstructed during separate scanning section is not reported in the literature [30–34]. A virtual organ is composed of a finite number of volume elements and its morphology and structure can be expressed through the geometric relationships between these volume elements. Cutting such a virtual organ requires relative positioning, which means that a body coordinate system needs to be established. When we tried to improve the accuracy and precision of an PAI oriented CSIs analysis, we utilized the PAI as the body coordinates of a 3D organ image and faced a new problem: since the multiplication of transformation matrices operating on a matrix (the coordinates of a point written as a column/row matrix) was not commutative [35], the iteration times to calculate the PAI were uncertain or even unpredictable. For a 2D image, to calculate its COM and PAI, only one translation and one rotation are needed, but for a 3D image, the number of iterations was an uncertain value and consequently required too long processing time.

Bearing in mind all unexplored clinical and technical aspects of the CSIs analysis in medicine, the specific purpose of the present paper is to introduce a novel method utilizing the PAI determination to extract the anatomically identically positioned CSIs obtained in different patients or obtained in the same patient during separate scanning sessions. In addition, we hypothesize that it is possible to construct an iterative relation during 3D reconstruction of a virtual organ by setting the control conditions. Specifically, by limiting the number of iterations, we aim to construct a unique body coordinate of a 3D organ. Hypothesis has been verified by reconstructing the CSIs of the first metatarsal bone of six participants.

## 2. Methods and Materials

### 2.1. Subjects and Imaging

The study was approved by the Ethical Committee of Fujian Normal University. The methods were carried out in accordance with the approved guidelines. The participants provided fully informed consent to participate in this study by signing a written consent form.

Two studies were carried out on the following experimental groups: (1) the right foot of one healthy male participant which was scanned twice, with an interval of 19 months between sessions; (2) the right foot of five healthy male wrestlers from a provincial sports school.

Before the scan, each participant's medical history was reviewed and each of them underwent X-ray imaging in order to exclude participants with conditions such as pathological changes, deformations, or injury of the foot. The feet were imaged using the CT scanner (Philips/Brilliance 64; KVP) operated at 120 KV. The scanning was conducted along both foot transects, from top to bottom. Participants were asked to remain in the standard anatomical position. The basic morphological features of the participants' first metatarsals, their position, and posture during scanning are given in Table 1.

### 2.2. Positioning of the Virtual Organ

The position of the organ's center of mass (COM) was obtained using the equations

(1) xc=∑i=1nxiρiΔV∑i=1nρiΔV,yc=∑i=1nyiρiΔV∑i=1nρiΔV,zc=∑i=1nziρiΔV∑i=1nρiΔV,

where (*x*_*c*_, *y*_*c*_, *z*_*c*_) is the set of coordinates for the organ's COM, (*x*_*i*_, *y*_*i*_, *z*_*i*_) is the set of coordinates for each volume element in the CSI, Δ*V* is the volume element, *ρ* is the density of the volume element, and *n* is the number of volume elements.

The rotation angle around the *x*-axis is

(2) $$\begin{array}{c} \alpha =\frac{1}{2}arctan\left(\;\frac{2\sum y_{oi}z_{oi}\rho_{i}\Delta V}{\sum y_{oi}^{2}\rho_{i}\Delta V-\sum z_{oi}^{2}\rho_{i}\Delta V}\;\right), \end{array}$$

where ∑*y*_*oi*_*z*_*oi*_*ρ*_*i*_Δ*V* represents the products of inertia rotating around the *x*-axis and ∑*y*_*oi*_^2^*ρ*_*i*_Δ*V*, ∑*z*_*oi*_^2^*ρ*_*i*_Δ*V* represent the moments of inertia.

The positional coordinates of the volume elements after a rotation by an angle *α* around the *x*-axis are

(3) xiαyiαziα=1000cos⁡α−sin⁡α0sin⁡αcos⁡αxioyio−yczio−zc+0yczc.

The rotation angle around the *y*-axis is

(4) $$\begin{array}{c} \beta =\frac{1}{2}arctan\left(\;\frac{2\sum x_{\alpha i}z_{\alpha i}\rho_{i}\Delta V}{\sum x_{\alpha i}^{2}\rho_{i}\Delta V-\sum z_{\alpha i}^{2}\rho_{i}\Delta V}\;\right), \end{array}$$

where ∑*x*_*αi*_*z*_*αi*_*ρ*_*i*_Δ*V* is the product of inertia after rotating around the *y*-axis and ∑*x*_*αi*_^2^*ρ*_*i*_Δ*V*, ∑*z*_*αi*_^2^*ρ*_*i*_Δ*V* are the moments of inertia.

The positional coordinates of the volume elements after a rotation by an angle  *β* around the *y*-axis are

(5) xiβyiβziβ=cos⁡β0sin⁡β010−sin⁡β0cos⁡βxiα−xcyiαziα−zc+xc0zc,

where (*x*_*i*_^*α*^, *y*_*i*_^*α*^, *z*_*i*_^*α*^) is defined as in (4).

The rotation angle around the *z*-axis is

(6) $$\begin{array}{c} \gamma =\frac{1}{2}arctan\left(\;\frac{2\sum x_{\beta i}y_{\beta i}\rho_{i}\Delta V}{\sum x_{\beta i}^{2}\rho_{i}\Delta V-\sum y_{\beta i}^{2}\rho_{i}\Delta V}\;\right), \end{array}$$

where ∑*x*_*αi*_*z*_*αi*_*ρ*_*i*_Δ*V* represents the products of inertia rotating around the *z*-axis and ∑*x*_*αi*_^2^*ρ*_*i*_Δ*V*, ∑*z*_*αi*_^2^*ρ*_*i*_Δ*V* are the moments of inertia.

The positional coordinates of the volume elements after a rotation by angle *γ* around the *z*-axis are

(7) xiγyiγziγ=cos⁡γ−sin⁡γ0sin⁡γcos⁡γ0001xiβ−xcyiβ−ycziβ+xcyc0,

where (*x*_*i*_^*β*^, *y*_*i*_^*β*^, *z*_*i*_^*β*^) is defined as in (5).

Processing according to (2)–(7) was performed iteratively. The process was completed when and only when *α* = 0, *β* = 0, *γ* = 0 (usual error margin 0.001). Since the PAI have no direction (whereas the coordinate axis does have one), the organ's posture varies after it has been positioned. We took the standard anatomical posture as a starting point. For instance, when the anteroposterior position of the foot was reversed after being positioned, we flipped the canvas horizontally by 180° in the vertical direction.

### 2.3. Reconstructing a Virtual Organ

The reconstructed CSI is isotropic:

(8) xi′=Roundxi−xcR,0R,yi′=Roundyi−ycR,0R,zi′=Roundzi−zcR,0R,

where *R* represents the CSI resolution (in this case, 0.5 mm; the logical slice distance is 0.45 mm, but to ensure reconstruction quality, we chose a greater resolution), (*x*_*i*_′, *y*_*i*_′, *z*_*i*_′) is the set of coordinates of the reconstructed (*x*_*i*_, *y*_*i*_, *z*_*i*_), and (*x*_*i*_, *y*_*i*_, *z*_*i*_) and (*x*_*c*_, *y*_*c*_, *z*_*c*_) are defined as in (1).

For CSIs along one axis, we obtain along the horizontal axis

(9) xi′=xip+Δ>xi>p−Δspaceotheryi′=yiotherspacexi′=spacezi′=ziotherspacexi′=space,

along the frontal axis

(10) xi′=xiotherspaceyi′=spaceyi′=yip+Δ>yi>p−Δspaceotherzi′=ziotherspaceyi′=space,

and along the vertical axis

(11) xi′=xiotherspacezi′=spaceyi′=yiotherspacezi′=spacezi′=zip+Δ>zi>p−Δspaceother.

In (9)–(11), *p* denotes a point on an axis, with *p* ∈ (0, ±*R*, ±2*R*,…). (*x*_*i*_′, *y*_*i*_′, *z*_*i*_′), (*x*_*i*_, *y*_*i*_, *z*_*i*_) and *R* are defined as in (8) and 0 < Δ < (1/2)*R*. These equations are used to reconstruct cross sections along a chosen axis.

## 3. Results and Discussion

Deductive reasoning shows that asymmetrically shaped and anisotropically distributed objects, such as human organs [36], have unique body coordinates [37]. The key issue in our study was to test whether the original morphology and structure of an organ will be preserved after it is reconstructed based on its unique body coordinates. Collectively, observed lines of evidence suggest that the proposed method utilizing PAI oriented 3D virtual organ reconstructions has a considerable potential to match CSIs obtained at different time points or from different participants.

**Figure 1:**
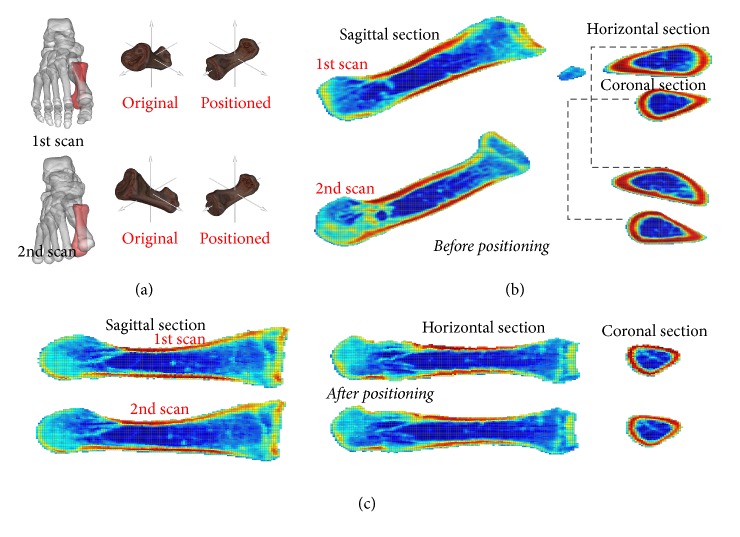
Standardized cross section images (CSIs) of the first metatarsal of the right foot. (a) Standardized body coordinate system of the first metatarsal. (b) Center of mass (COM) on the coronal, horizontal, and sagittal plane before positioning. (c) COM on the coronal, horizontal, and sagittal plane after positioning. Equation (1) is used to calculate the COM of the metatarsal. Equations (9)–(11) are used to establish the three sections of the scanned posture (with the COM of the metatarsal on the planes); see panel (a). Equation (1) is used to calculate the COM of the metatarsal and (2)–(7) are applied successively to position the metatarsal; (8) is used to reconstruct the CSIs (panel (b)), while (9)–(11) are used to establish the three sections after they are positioned (with the COM of the metatarsal on the planes); see panel (c). After the first scan, positioning of the first metatarsal was completed in two iterations, first by rotating about the *x*-, *y*- and *z*-axes by 34.25, −10.31, and 22.48 degrees, respectively, and then by 0.09, 0.03, and 0.00 degrees, respectively. Also, after the second scan, positioning of the first metatarsal was completed in two iterations, by rotating about the *x*-, *y*- and *z*-axes first by 38.34, 14.92, and 5.86 degrees, respectively, and then by −0.04, 0.00, and 0.00 degrees, respectively. The COM of the first metatarsal was 69.55, 150.48, and −111.94 mm, in the first scan and 70.30, 91.72, and −168.04 mm in the second scan. The origin of the coordinate system was set at the organ's COM.

In this section, we use the term “standardization” to refer to establishing a coordinate system based on the organ's PAI and to subsequently setting the organ's COM as the origin of the coordinate system. As can be seen in Figures 1(a) and 1(b), the participant's position and posture were significantly different in the two scans. Based on the body coordinate system created according to the PAI of the virtual organ, we reconstructed the two scan CSIs of the metatarsal along the same axis (Figure 1(c)). The ability to perform such an operation is of significant importance for clinical practice, as with our method clinicians can accurately position the lesions and observe changes therein for the same cross section plane, even if the images were obtained in different scanning sessions. This enables a more direct comparison, potentially resulting in a more precise and effective diagnosis as changes can be detected unambiguously when the same location has been scanned multiple times. Our method might therefore lead to a reliable predictor in clinical diagnosis, where the visualization of the organ body coordinate system can enable a qualitative diagnostic analysis of CSIs taken for observed organ with improved quality and reliability.

**Figure 2:**
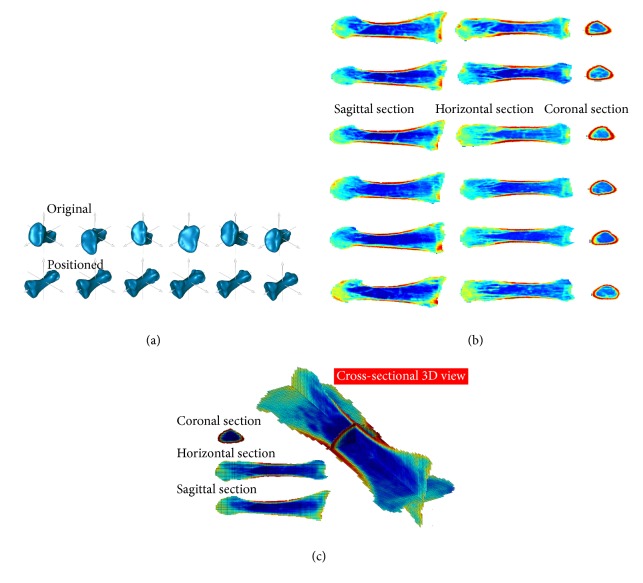
Merging of the cross section images of the first metatarsals of the left foot. (a) Standardized body coordinate system of the first metatarsal of the left foot. (b) Standardized geometry of the first metatarsal of the left foot. (c) Center of mass on coronal, horizontal, and sagittal planes after the CSIs were merged. The processing method is the same as that of the first metatarsal in Figure 1.

Going beyond enabling a more direct comparison between two images, our method opens up a route to create an average CSI over several images. Namely, a virtual organ consists of a finite number of cross sections, which means that modelling of an average organ should start from average CSIs. To this end, a second part of the current research was conducted to analyse CSIs dataset of the feet of six volunteers. The first metatarsals of their left foot were selected for standardization. After positioning, the CSIs were merged (Figure 2). Evidently, the positions and postures of the first metatarsals are different among the participants, as are the morphology and structure for each individual bone (Figure 2(b)). Clinically, numerous indices of healthy people are considered as diagnostic standards (e.g., temperature, body mass index, blood pressure, and bone mineral density) [38]. This is also true for CSIs. It is therefore necessary to model an average CSI based on differences between individuals and on the requirements for a diagnosis. After the length and width vertical to the principal axes of a CSI were rated by percentages, three average cross sections of the first metatarsal from the six volunteers were calculated (Figure 2(c)). This figure shows that an average cross section of the same organ from different participants can be reconstructed. In practice, an attempt to compare the CSIs of an organ from different patients may lead to the misinterpretation of the radiological findings if the extreme differences between the same structures from different patients exist. However, proposed approach provides the promising opportunities to standardize the protocols for the radiological examinations and compare the outcomes observed at different time points as well as the results obtained in different studies. It is justified to assume that although the variations exist, it is possible for precisely defined CSI to determine average anatomical outcomes. Furthermore, it is also reasonable to stipulate that significant differences could be observed at the same CSI if healing or pathological processes between two time points take place which can influence the correct PAI establishment. Thus, for pathological process the whole organ or sometimes even surrounding anatomical structures should be taken into consideration during 3D reconstruction.

When the scanning axis of the equipment is fixed, the same holds true for the coordinate system of the scanning area. The body coordinate system of an organ is then determined by the position and posture of the participant when being scanned. At present, attempts are made to immobilise patients reproducibly in fixed position by using stereotactic frames [39]. An ideal approach is to develop a method for constructing a body coordinate system regardless of scanning posture or cutting axis. Our study offers such a method. We reconstructed CSIs of the first metatarsal along any axis (see supplementary animation file (available here)), offering a direct example to illustrate the power of our approach. After the organ is reconstructed based on its unique body coordinates, its original morphology will be retained, as well as its structure. The CSIs along arbitrary axes reflects the structure of an organ from different angles and different positions. Using the present method, the body coordinates can rotate on the organ, so that we can observe cutting or scanning section along arbitrary axes, as a virtual organ retains its geometric invariance through processes such as rotation or translation [40].

The results showed that when we utilized the COM and three PAI of a 3D organ as its body coordinate, we could successfully register images from the same participant's different times' scans or from the same organ of different participants, suggesting that an average CSI can best serve for medical education. The invariation of an organ to its coordinate transformation can be verified by Target Registration Error tests, with an accuracy of submillimeter.

To calculate the PAI of an organ, we need to extract the organ (from each cross section). This work is done manually, which might affect its reproducibility, and represents a limitation of this study. When reconstructing the pixels that comprise an organ, the rotation may lead to some “free points,” which do not belong to section *i*, section *i* − 1, or section *i* + 1. We will investigate how to identify and determine these “free points” in future work.

The clinical significance of presented approach lies in the fact that after one scan session, a clinician can study the morphology and structure of a lesion using CSIs along arbitrary axes. Clinician can follow over time morphological and structural changes in the same plane of a lesion, using different scans from the same patient. In this way, the rate of misdiagnoses related to CSI will be reduced. The CSIs are from the same position and posture, and the errors are controlled within submillimeter distance. In addition, it is believed that proposed method can be potentially used for the customization of orthopaedic prosthesis. For example, after positioning and mirroring the uninjured side of an organ, we can design and develop prosthesis for the injured side. This prosthesis is the closest in properties to the uninjured side, and we may call it specially custom-made for the individual. Again, it has been demonstrated that the presented method has a potential to overcome a critical hurdle of not-cutting principal axes in defected virtual organ of injured side.

## 4. Conclusions

In summary, we demonstrated that the construction of human virtual organs by stacking CSI scan enables their positioning independent of the scanning or cutting axis. In this manner, the unique body coordinates of an organ are established based on their PAI. As a result, CSI reconstruction along arbitrary axes of the organ's body coordinates becomes a reality. Clinically, this method can be widely applied to observe CSIs from multiple scans in the same position and posture and consequently avoid radiological misdiagnosis.

## Figures and Tables

**Table 1:** Basic information for the first metatarsal.

Participant	Volume^a^	Area^b^	Position^c^	1st rotation^d^	2nd rotation^d^
(1)	12,359.49	3,674.19	(187.82, 90.35, 162.21)	(38.80, −15.84, −3.13)	(−0.02, 0.00, 0.00)
(2)	15,135.75	4,156.17	(194.25, 105.35, −185.41)	(25.21, −25.58, −10.52)	(−0.15, 0.03, 0.00)
(3)	12,517.31	3,732.70	(224.86, 108.32, −215.00)	(−42.04, 4.98, 22.07)	(1.63, −30.84, −0.02)^*∗*^
(4)	16,644.79	4,409.09	(183.93, 101.91, −191.81)	(36.60, −37.49, 0.07)	(−0.05, 0.00, 0.00)
(5)	17,006.04	4,523.55	(208.98, 107.08, −208.79)	(30.06, −23.61, −0.57)	(−0.01, 0.00, 0.00)
(6)	18,133.94	4,591.07	(228.24, 103.84, −200.65)	(43.94, −13.99, −15.57)	(−0.11, 0.03, 0.00)

^a^mm3, ^b^mm2, ^c^mm, and ^d^deg. ^*∗*^The  3rd  rotation  was  (0.01, 0.00, 0.00).

## Supplementary Materials

Supplementary Materials Supplementary video files: (1) r-l-a.flv video recording changes on the axial plane of the first metatarsal boundaries from the same participant's left and right foot; (2) r-l-c.flv video recording changes on the coronal plane of the first metatarsal boundaries from the same participant's left and right foot; (3) r-l-s.flv video recording changes on the sagittal plane of the first metatarsal boundaries from the same participant's left and right foot; (4) f-s-a.flv video recording changes on the axial plane of the right foot first metatarsal boundaries from the same participant's first and second time; (5) f-s-c.flv video recording changes on the coronal plane of the right foot first metatarsal boundaries from the same participant's first and second time; (6) f-s-s.flv video recording changes on the sagittal plane of the right foot first metatarsal boundaries from the same participant's first and second time.
